# Applying Kumpfer’s resilience framework to understand the social adaptation process of the trailing parents in China

**DOI:** 10.1186/s12877-024-05170-3

**Published:** 2024-07-09

**Authors:** Yuehui Yu, Depeng Li, Yun Xia

**Affiliations:** 1 School of Public Administration and Policy, Renmin University of China, Beijing, 100872 China; 2 School of Marxism, Renmin University of China, Beijing, 100872 China

**Keywords:** Trailing parents, Social adaptation, Resilience, Social support

## Abstract

**Background:**

Trailing parents, a distinct group emerging from China’s rapid social change and urbanization, are experiencing migration in old age, posing challenges for their social adaptation. Existing research has mainly focused on the hardships faced by this group, but few studies have focused on how they cope with change and achieve some degree of successful social adaptation. This study aimed to understand the coping and social adaptation process of trailing parents in China.

**Methods:**

This study used a qualitative research approach. A total of 24 trailing parents were invited to participate in a semi-structured interview and share their experiences and efforts to cope with the many challenges. Kumpfer’s resilience framework was used as the theoretical framework for the study design, data collection, and data analysis.

**Results:**

This study identified several intra-family and community stressors that trailing parents may face when moving to a new environment and uncovered five key resilience characteristics that may be triggered or fostered in the presence of these stressors, including physical fitness, psychological stability, open-mindedness, learning ability, and nurturing hobbies. Individuals with resilience traits have been observed to engage in positive cognitive processing and transform the new environment. Consistent with Kumpfer’s resilience framework, this study revealed the dynamics of the stressors faced by trailing parents in the new environments, the role of resilience characteristics, and the critical influence of social support in shaping the interplay between the individual and the environment that enabled them to adapt positively.

**Conclusions:**

This study highlights the importance of fostering resilience traits and leveraging positive coping mechanisms to facilitate a smoother adaptation process for trailing parents. Meanwhile, there is an urgent need to focus on creating opportunities that strengthen their social support networks.

## Background

Chinese society is characterized by ‘high mobility’ due to the economic boom and accelerated urbanization [1]. According to the seventh national census, the migrant population has reached 376 million, accounting for 26.0% of the total population. Most migrants move from rural to urban areas in search of employment opportunities [2, 3]. As part of China’s household registration (hukou) reform, large numbers of rural young people have obtained urban hukou and citizenship by working in cities [4].

In recent years, a new group of migrants has emerged as a by-product of the overall picture of migration and urbanization. As young people settle and work in cities, a group of older people, mainly unemployed or retired parents, choose to migrate to live with their adult children and help with family responsibilities. They are not fully ‘settled’ because they do not have a hukou and are not entitled to citizenship there. Instead, their hukou and associated social benefits, such as health care and pension, remain in their place of origin. As a result, they have a migratory character, routinely moving back and forth between the children’s city and their own hukou places. This group of people is known as ‘trailing parents’ [5], ‘drifting elderly’ [6], ‘floating elderly’ [7], ‘older migrants’ [8] or ‘floating grandparents’ because most of them migrate to take care of grandchildren [9]. They are collectively referred to in this study as ‘trailing parents’.

There are more than 18 million trailing parents in China, and the number is still growing [10]. Migration with adult children is a reflection of the Chinese family ethics [11, 12]. Older parents feel a strong responsibility to support their adult children, so they choose to start a challenging life in an unfamiliar city. The trailing parents have the vulnerability characteristics of both ‘migrant’ and ‘old’. Migration means a break with the original social network, so migrants often face challenges in terms of lifestyle, values and interpersonal relationships [6, 13]. At the same time, behaviors and ways of thinking are more ingrained in older people, making them more difficult to adapt to change and more vulnerable to crises in their daily lives [14].

Many studies have focused on the issue of social adaptation of the trailing parents, showing that they face difficulties such as discomfort with the local environment, social isolation, interpersonal conflicts and difficulties in accessing health care [6, 10, 12]. Underlying these difficulties are the intersections of institutional exclusion and cultural segregation [8]. The joy of reuniting with children and grandchildren is largely diminished by the difficulties of social adaptation [15]. In general, the trailing parents have a low sense of self-identity and a poorer overall quality of life [8, 13].

In addition to the overall negatives, a number of studies have drawn attention to the heterogeneity of the trailing parents, finding that levels of social adaptation vary between individuals and change over time [16, 17]. Although confronted with difficulties, some are able to adapt continuously, integrate positively into the new environment, rebuild social networks and confidence in life, and thus maintain a good level of psychological well-being. Meanwhile, social adaptation is both an outcome and a process. Over time, trailing parents become familiar with the new environment and adapt their behavior [12].

Although social adaptation as an outcome has been studied extensively, current studies have not uncovered the process by which the trailing parents explore, interact with, and ultimately achieve social adaptation. What are the main stresses that trailing parents may face when they first enter an unfamiliar social context? How do they cope with these challenges and gradually adapt? What can we learn from people who have successfully adapted? This study applies Kumpfer’s resilience framework [18] to analyze the experiences of trailing parents, in particular to understand how they build resilience in their social adaptation process, in order to gain more positive insights for further promoting the psychological well-being of the trailing parents in China.

Kumpfer’s resilience framework considers both factors and processes that enable people to become adaptable. When faced with stressors, the environmental stimulus leads to person-environment interactions that strengthen resilience characteristics. The resilience characteristics then interact dynamically with the environment, leading to different outcomes [18]. This framework has been widely used to describe the resilience process of children, adolescents, caregivers of disabled people, patients with special conditions and working mothers [19–21]. This study applied Kumpfer’s resilience framework to trailing parents in China. It first focused on the stressors that trailing parents may face when encountering an unfamiliar context, and then analyzed the resilience characteristics and the person-environment interaction process in which trailing parents’ multiple resilience characteristics continually contribute to shaping proactive outcomes.

## Methods

### Study design

This study aimed to explore the social adaptation process of the trailing parents in the context of China’s rapid urbanization, and therefore adopted a qualitative research approach. A total of 24 trailing parents were invited to participate in a semi-structured interview and talk about their experiences, stress and efforts to cope with the many challenges. Kumpfer’s resilience framework was used as the theoretical framework for the study design, data collection and data analysis.

### Study setting and participants

In qualitative studies that aim to capture people’s experiences and perceptions, the context in which they are situated is important [22]. As trailing parents routinely travel between their children’s cities and their hometowns, their perceptions and narratives of the migrant experience and social adaptation may be influenced by their current living context. Those who live in cities and those who return home temporarily may have different feelings to share. Therefore, in order to ensure the appropriateness, relevance and diversity of the information that participants would provide, this study was conducted in both the inflow city and the outflow areas. Specifically, we interviewed 13 trailing parents living with their adult children in Beijing, and another 11 trailing parents who had temporarily returned to their hometowns in Henan and Hubei provinces.

In this study, we used purposive and snowball sampling to recruit trailing parents. We identified suitable participants with a migration experience of more than one year, during which time they regularly travelled between home and the host city. In addition, they agreed to participate in the interview and were able to communicate well. Older adults who had migrated for less than a year were excluded because they were still in the process of adapting. Those who had fully settled in the city, even without a hukou there, were also excluded because they no longer migrated, which distinguished them from the mainstream of trailing parents. The sample size was determined on the basis of information saturation [23, 24].

### Data collection

A semi-structured interview guide was developed based on Kumpfer’s resilience framework, literature and the study objectives. All 24 interviews were conducted by experienced qualitative researchers from the study team between August 2022 and May 2023. A relatively long period of data collection gave the researchers plenty of time to get into the minds of the trailing parents, to think critically about their experiences and to improve the quality of subsequent interviews.

Initially, a pilot study was conducted with two trailing parents to evaluate the guiding questions. At this stage, we also interviewed their adult children to refine the interview guide. The researchers then conducted interviews based on the refined guide. The interview questions were open-ended. Topics included: (1) basic information, including participants’ demographics and other relevant information about their family; (2) trailing parents’ experiences of migration and living in the new environment; (3) individual difficulties and stressors; (4) their responses and coping strategies; and (5) highlighted efforts to overcome challenges during the adaptation process.

The interviews in Beijing were conducted in the parks or outside the off-campus training institutions because it was easier to approach the participants there. After school, it is common for them to take their grandchildren to the parks or off-campus institutions for extracurricular activities, so we had plenty of time to conduct the interviews on these occasions. The interviews in Henan and Hubei provinces were conducted by home visit, as they were temporarily back in their hometown. During the interviews, the researchers observed the participants’ non-verbal behavior and living environment to gain a deeper understanding of them. Each interview lasted between 40 and 60 minutes. The interview recordings were compiled and transcribed by the interviewers shortly after each interview. The researchers also wrote interview notes, memos and observation records in a timely manner.

### Data analysis

In this study, we used a thematic analysis approach to analyze the data through a systematic and iterative process. In line with Kumpfer’s resilience framework, we coded the interview texts into different themes, including intra-family and community stressors, resilience characteristics, person-environment interaction process and social support in the resilience process. To ensure the reliability, the data analysis began at the time of data collection. Each transcribed text was manually analyzed independently by two researchers. The study team met regularly to discuss the codes and finalize the themes for characterizing the resilience of trailing parents. Throughout the process, the study team supported the work with ongoing memos and tables [25]. Information saturation was also monitored as needed.

### Data trustworthiness and ethics

To ensure the trustworthiness of the data, we continuously considered how to reduce the impact of subjectivity on the results [26, 27]. Firstly, all interviewers were well trained and had sufficient experience in qualitative research. We tried to maintain a neutral attitude when communicating with interviewees and took particular care not to influence them with our opinions. Secondly, non-verbal behavior, living environment and other contextual elements of the interviews were noted to provide corroborating evidence. Finally, peer review techniques were used among the researchers to reach agreement on coding and themes.

This study was conducted in accordance with the Declaration of Helsinki. Ethical approval was obtained from the Faculty Academic Ethics Committee. All participants gave informed consent after receiving a full explanation of the study. We have tried to be sensitive to participants’ feelings. If they felt uncomfortable during the interview, they could withdraw. In fact, these trailing parents were happy and willing to share their experiences of living in cities. By sharing, they gained a greater sense of self-efficacy.

## Results

This study involved 24 participants aged over 60 years (Table 1). Of these, 18 were women and 6 were men; 10 were over 70 years old. They migrated for a variety of reasons, the most important being to look after grandchildren (21/24), followed by to share household chores (16/24) and to reunite with adult children (15/24). Most of the following parents interviewed had migrated alone (16/24), leaving their spouses at home. Table 1 also shows the socio-demographics of participants in Beijing and other areas separately. Although the participants were in different locations at the time of the interview, there were no obvious differences between them.

**Table 1:** Characteristics of the trailing parents in the qualitative study

Classification		Participants in Beijing	Participants in Henan and Hubei	Total
Gender	Male	4	2	6
	Female	9	9	18
Age range	60–69 years old	8	6	14
	≥ 70 years old	5	5	10
Reasons for migration	Look after grandchildren	12/13	9/11	21/24
	Reunite with adult children	9/13	6/11	15/24
	Share household chores	9/13	7/11	16/24
Migrate with a spouse	Yes	5	3	8
	No	8	8	16

When the trailing parents migrated to a new context, they encountered both intra-family and community stressors that activated their adaptation and resilience-building process. Their resilience characteristics helped to facilitate the person-environment interaction process and generated positive social adaptation outcomes. Social support was helpful in accelerating the process of building resilience. Table 2 summarizes the main categories identified in this study and the elements of each category. Viewing social adaptation as a process, these categories interacted with each other dynamically.

**Table 2:** Summary of the categories of the study

**Experiencing stressors**	Intra-family stressors	Guest identity Heavy housework Role stress Intergenerational conflict Guilt about distant loved ones
	Community stressors	Unfamiliar environment and lifestyles Social disconnection and isolation
**Resilience building** **&** **Social adaptation**	Resilience characteristics	Physical fitness Psychological stability Open-mindedness Learning ability Hobbies and interests
	Obtaining social support	Intra-family support Support from friends and neighbors
	Person-environment interactional process	Positive cognitive processing Transforming the new environment

### Experiencing stressors

As newcomers, the study participants saw themselves as ‘guests’ in the family and ‘outsiders’ in the community. They faced stressors within the family and the community.

#### Intra-family stressors

Prior to migration, these study participants acted as a central hub in their original family, linking great-grandparents, their spouses, other children and grandchildren. In the new place, their main role is to care for grandchildren and even adult children, prioritizing these relationships, which has led to a disruption of the original family relationships and old living arrangements. This inverted relationship places older people in a stressful context, especially for those who have migrated alone.

##### Guest identity and heavy housework

In the early days of migration and living with adult children, the study participants saw themselves as guests. But unlike normal guests, who are treated well, they have to work without pay and without end. They were like nannies, doing all the household chores such as childcare, cleaning, cooking and many others. The heavy work interfered with their daily lives, caused great physical strain and kept them in a state of living for others, with no time for themselves.

*I used to have a lot of free time to play cards with my friends. Now, in this place, I don’t have any time for myself. It is really hard to be a trailing parent. But if I don’t, my son and daughter-in-law will be too much of a burden…I do almost all the family chores, I feel like a nanny, but unpaid…They (the adult children) come home from work and spend time with their children. At that moment I feel like an outsider or a guest. (No.1)*

Compared to less developed areas, the average living space in big cities is smaller. In many cases, the young family could not provide an exclusive room for the trailing parents, so they had to share a room with their grandchildren or even sleep in the living room. The living conditions were a constant reminder that they were not full members of the family, but temporary ‘guests’ who travelled between the city and their hometown.

*In my hometown, I lived in a spacious house of more than 200 square meters. Here it’s too small and cramped. I don’t have my own room, and there’s no stability. Every year I travel back and forth between Beijing and my hometown. I do not belong here. I am a guest. When my grandchildren grow up, I won’t stay here anymore. (No.21)*

##### Role stress and intergenerational conflict

Retirement should be the end of certain social responsibilities, not the beginning. However, in order to ease the burden on adult children, the study participants took on new responsibilities as caregivers after retirement. Some were under great pressure to change roles and learn new skills, such as cooking and babysitting. Their adult children had high expectations of their new role and, as new learners, they were not perfect.

*It was very uncomfortable when I first arrived, especially having to cook. At home I did not have to worry about food, we had a canteen. Here I have to cook for every meal. I have tried very hard, but they (the adult children) are still not satisfied. (No.12)*

The study participants saw themselves as respectable parents in the ‘new’ family, but other family members saw them as caregivers. Differences in role perceptions had led to many intergenerational conflicts. As parents, they wanted to be listened to and involved in family decisions. But their opinions were often ignored, leaving them frustrated and angry.

*The birth rate is falling, so sooner or later the house price will fall. Last year I suggested they sell the extra flat, but my daughter-in-law was not happy. Now they want to sell it, but they can only get a much lower price…No one listened to me. I feel angry when I raise this point. They have lost a lot of money, much more than they can earn for a year. I don’t have a voice at home, for many things. (No.5)*

##### Guilt about distant loved ones

Prolonged separation posed a major challenge to maintaining close relationships with family members in hometowns. As trailing parents, the study participants have faced a caregiving dilemma. On the one hand, they have the responsibility of caring for grandchildren and adult children in the place where they migrate, and on the other, they have parents and spouses who are unable to migrate with them. As older adults, they felt more empathy for their own parents and were therefore constantly burdened with guilt for not being able to fulfil their filial duties. Especially when they heard that a loved one was ill or something else had happened, they felt a strong sense of guilt and regret that they could not be with them in time.

*I have parents in my hometown, both in their 80s, and my mother’s eyesight has not been good over the years. I am always worried about their health and safety…The whole life is a state of worrying about both sides. When I was in my hometown, I worried about them in Beijing, and when I am here, I worry about my old parents in my hometown, even feeling guilty that I cannot take care of both sides. (No.22)*

#### Community stressors

Moving to a new community meant not only an unfamiliar spatial structure for the study participants, but also a sense of insecurity. Their sense of belonging was disrupted by the dualistic dichotomy of identity between local and foreign. The exclusivity of public services or resources associated with hukou status and citizenship also exacerbated the isolation they felt in the community.

##### Unfamiliar environment and lifestyles

Inter-regional migration brings changes in culture, language and customs. The new communities have the social norms of a specific urban context, most of which use standard Mandarin as daily language. However, most of the study participants were accustomed to speaking a dialect and had formed relatively fixed values, living habits and social codes of behavior. In the new community, they faced language barriers, difficulties in adapting to the culture and lifestyle, and a sense of alienation.

*I don’t speak Mandarin very well yet. I prefer not to communicate with those who speak Mandarin, they would look down on us. I do not like the food here. The climate is also hard to get used to. I get sick every time I come here. (No.17)*

The study participants perceived the new urban community as dense, complex, more mobile but less interactive. Unlike young people who may experience a sense of novelty when moving to a new place, older people tended to experience complex feelings of meaninglessness, fear, anxiety and self-blame for not performing well. Negative feedback further discouraged them from taking initiatives to adapt.

*I easily got lost in the city. When I first came here, I was afraid to go downstairs. I stayed in a room for a week before I went down, living like a prisoner…I didn’t know how to use the vending machine downstairs and tried half a dozen times by myself, stopping when someone walked by for fear of the jokes. (No.9)*

##### Social disconnection and isolation

Without a support network of relatives and acquaintances, the study participants felt socially isolated in the community. Due to cultural and language barriers, they lacked opportunities to make friends and were unable to integrate or receive community support like local residents. They found it difficult to cope with daily chores, emergencies or health problems. In most cases, they could only rely on their adult children for support. But the children were busy working and had no time to notice their needs.

*The locals get together and talk about everything, while the foreigners always feel rusty and talk very little. We don’t know each other well. For the first few months, I relied on the children for almost everything. Whatever the problem, all I can do is ask them for help. They obviously feel they have too much on their plate. (No.19)*

In addition to informal social support, the study participants were also excluded from the formal support associated with local citizenship. Because of the hukou system in China, barriers to health insurance and other public services have not been fully removed, leaving a significant proportion of migrants with inadequate access to social services in their place of residence. Their leisure, health and psychological needs were not being met as well as those of the locals. The internalized sense of marginalization made them even more reluctant to reach out.

*The community notifies people over 60 to go for free medical check-ups and flu vaccinations. But only those with local hukou are eligible. They were vaccinated and given small gifts. I felt really bad that we outsiders had nothing. We really don’t belong here. We are here for the grandchildren, not for ourselves. (No.16)*

*We get little reimbursement for off-site medical care, so the cost of medicine is much higher. Last year my tooth hurt and I suffered a lot. I persevered until I returned to my hometown to get it fixed. There I feel safe and not taken advantage of. (No.8)*

### Resilience building

#### Resilience characteristics

Resilience characteristics refer to positive internal personal qualities that trigger the resilience process and interact with the external environment. In general, those well-adapted study participants were able to acquire, possess and maintain good physical fitness, psychological stability, open-mindedness, active learning ability and specific hobbies. These played a crucial role in achieving positive social adaptation outcomes.

##### Physical fitness

Study participants who were in good physical condition were better able to adapt when they migrated. Physical fitness gave them a good physiological basis for coping with stressful events and prevented the negative psychological emotions internalized from somatic illness. Independence also made it easier for them to develop confidence.

*It is important to stay healthy. We are here to take the burden off them (the adult children), not to add to it. I exercise every morning and I am healthy. I can do all kinds of housework and also help the neighbors. I feel that I am worthwhile. (No.18)*

##### Psychological stability

Study participants who were optimistic, outgoing and content were able to reduce perceived stress through positive cognitive processing, making connections, sharing experiences and seeking support. It was easier for them to achieve a greater sense of material happiness and satisfaction, and to stabilize their mood through self-affirmation.

*It’s the good mentality that nourishes people. I don’t complain. I seldom get angry or have my mood suppressed. People get sick easily because of mood problems…No one wants to spend time with a person in a bad mood, listening to their complaints. I’m an optimistic person, so it’s easy for me to make new friends (No.3)*.

##### Open-mindedness

When migrating to an unfamiliar community, all study participants felt a reduced sense of control and showed social withdrawal. The initial period of maladjustment was shorter for the open-minded. They were able to adapt their psychological state more quickly and showed a greater desire to explore and change the environment. They were self-motivated to take steps to improve their lives, especially to develop new social relationships and to make full use of community resources.

*When you’re in a new environment, don’t think about the old one. Be open to new things and you will find that there are many good things about the big city. It gives me a lot of opportunities to meet new people from all over the country, try new things and improve myself. Although it is stressful, I have gained a lot here. (No.15)*

##### Learning ability

The ability and willingness to learn was important in speeding up social adaptation when people entered a new environment. Participants with good learning skills found it easier to remember new places and routes, master new equipment and assimilate a new language system. Through self-directed learning, they were able to understand the heterogeneous cultural system, increase their problem-solving skills, personal competence and sense of autonomy, all of which contributed to their satisfaction with their ‘new’ life.

*There are lots of new things to learn and it’s a struggle not to. When I first came here, my children taught me how to use map apps, taxi apps and how to shop online. They are actually very easy and quick to learn. Having mastered these skills, I feel like I’m really a city person now, and it’s so convenient. (No.20)*

##### Hobbies and interests

The study participants benefited greatly from cultivating and displaying hobbies, as it enriched their personal lives, compensated for monotonous repetitive work and boring solitary routines, and prevented them from being trapped in a mere ‘babysitting’ role. They created a unique social network based on their hobbies and interests, and realized their self-worth through daily interactions with others, which supported social adaptation.

*I have learnt to recite in the community and I have made many friends through it. We give each other positive feedback. I feel quite good and fulfilled. (No.13)*

#### Obtaining social support

Social support played an essential role in the social adaptation process. Social support for the study participants came mainly from family and communities, providing emotional support, information and practical help. This support had eased the burden of adaptation, provided emotional comfort and sustenance, and given them more resources and skills to cope with life transitions.

##### Intra-family support

Initially, many study participants faced strong barriers to integration into local social life due to shyness and unfamiliarity. Family support had played an important role in expanding their social network. For example, the younger generation organized some weekend gatherings that brought together several families of the same structure. This had not only strengthened family ties, but also provided opportunities for older adults to meet and socialize with others like themselves.

*When I first came here, I didn’t know anyone and didn’t talk to anyone. My son took me to his friend’s child’s birthday party and I met the child’s grandmother, and then we often meet and hang out after the children go to kindergarten. (No.16)*

In addition to connecting them to new social networks, family members could also reduce their subjective stress by actively ‘doing the little things’ for them, such as buying small gifts, noticing and praising their progress, and relieving them of household chores. Even small positive actions could have a big impact on their psychological relief, as these kind behaviors allowed this sensitive group to be more positive in their cognitive processing and avoid over-interpreting themselves as ‘guests’ or ‘caregivers’.

*My son said he wanted to hire an hourly worker to help me. He did not want me to be exhausted. I can take care of everything in the family. It’s enough for him to be considerable…He also buys me gifts every time he goes on a business trip. We older people don’t care too much about material things, but love is important. (No.14)*

##### Support from friends and neighbors

Given the hukou system in large cities, the study participants felt that becoming a citizen was unrealistic, even for those who had been migrants for several years. Therefore, most of them participated in activities initiated by other trailing parents in their communities. This unique neighborhood relationship continued to help them build self-efficacy. There were many things that could make them feel good about themselves in the community, such as learning how to get along with adult children, getting timely information about market discounts, and acquiring different cooking skills, especially if these efforts were noticed by adult children.

*We people from different provinces have a good time here and we’re all very united. For example, they would call me first if a supermarket was offering discounts. When they (adult children and grandchildren) go to work or school, we take turns visiting each other and trying new dishes together. My family loves the new food. (No.7)*

Despite the many difficulties they faced, the participants in the study said that they rarely got together to complain because it would bring out their common hardship and exacerbate the negative emotional experience. Instead of whining together, they offered each other positive emotional support by persuading the person to see the positive side of things. For example, when a family conflict arises, they flatter each other about their adult children’s successes and filial piety in many things, or praise them for taking good care of their grandchildren. They would also offer advice on specific difficulties.

*Sometimes I feel really terrible and want to go back to my hometown, especially after a conflict with the children. My friends would tell me that we can live here, in the big city, because our children are promising. They (the adult children) have difficulties in their work and we, as parents, should be more understanding. Without their company, I would have been inclined to be negative. (No.9)*

With the help of other trailing parents, the study participants were able to update their knowledge of the community culture through continuous observation, memory and active experimentation. New things were gradually transformed from unbearable burdens to pleasurable experiences in the new community. The expansion of social support networks reduced their dependence on families and nostalgia for distant loved ones. Although still trapped in a state of migration, their sense of drift had been reduced.

#### Person-environment interactional process

Family and community stressors stimulated person-environment interactions and strengthened resilience traits in the trailing parents who participated in this study. These characteristics then contributed to cognitive and behavioral changes that ultimately led to positive outcomes.

##### Positive cognitive processing

The participants in the study were not passive recipients of stress. Instead, they had the ability to filter and reinterpret stress. Their resilience characteristics created a protective mechanism to reduce stress levels in cognitive processing. They tended to put a positive spin on things. For example, although their feelings were often neglected by adult children, some were able to shift their perspective to focus on the good. They tended to attribute negative experiences to objective conditions that could not be resisted, and their suffering was not unique. They felt a strong desire to strive, learn, adapt, contribute and integrate in the big cities. In this way, they avoided magnifying the negative effects of stressful events and returned to psychological stability.

*It’s true that there are communication barriers with children, but they are filial. There are cognitive differences between the generations that we have to recognize… Nowadays, everyone talks about ‘active ageing’. We take on more family responsibilities so that our children can focus more on work and contributing to the city. In this sense, we are also valuable to society. (No.9)*

Their positive cognitive processing also involved a shift in role perceptions. Over time, the study participants came to accept their role as ‘helpers’ rather than as ‘traditional Chinese parents’ who, in the filial culture, must be fully respected and obeyed. As they did so, their dissatisfaction and hostility with the loss of authority largely diminished. They were able to adopt behaviors appropriate to their new role, taking the initiative to maintain harmonious family relationships and avoid conflict.

*There’s an old Chinese saying: ‘If you don’t listen to older people, you will suffer’, because they have a lot of experience. But we have to admit that the environment has changed. We don’t have much experience in the urban context. It’s not appropriate to continue to guide them with authority as ‘parents’. Now I unconditionally support their decisions. I just help them with the family chores. (No.6)*

##### Transforming the new environment

When resilience characteristics were activated by stressors, the study participants were self-motivated to transform the new environment and regain a sense of control over their lives. For example, to resist the alienation of city life, they would take some initiatives to create a more connected and meaningful community life, even breaking the segmentation between locals and migrants. They discovered their strengths and values and contributed actively to the community. Through genuine commitment, they gradually transformed the alienated community into a friendlier one, increasing the bonds of intimacy and trust, and gaining a sense of accomplishment in the process.

*I used to be a teacher. So here I am tutoring two grandchildren after school. When I saw that other grandparents had big problems with tutoring, I suddenly had an idea. I went to the community committee, persuaded them to reopen the small library and volunteered to tutor the children after school. The committee thought it was a great idea and agreed. Now people send their grandchildren here to do their homework, even the locals. They show me respect and love. I feel fulfilled here. (No.10)*

The study participants also showed a great deal of tactful wisdom in changing the family atmosphere, much of it learned from their peers. Rather than taking direct action against many family stressors, they sought to deconstruct the conditions that create or exacerbate those stressors. For example, they might have different ideas about how to raise grandchildren, which could easily lead to intergenerational conflict. In this case, they looked for more subtle and indirect ways to build consensus within the family.

*They (the adult children) are too strict about their daughter’s education, it’s not good for her. Talking to them directly might lead to an argument. I tried to find some parenting videos and shared them on the family WeChat group (a social media). They watched the videos and slowly agreed that they shouldn’t be so strict. (No.23)*

### Dynamic interaction

**Fig. 1:**
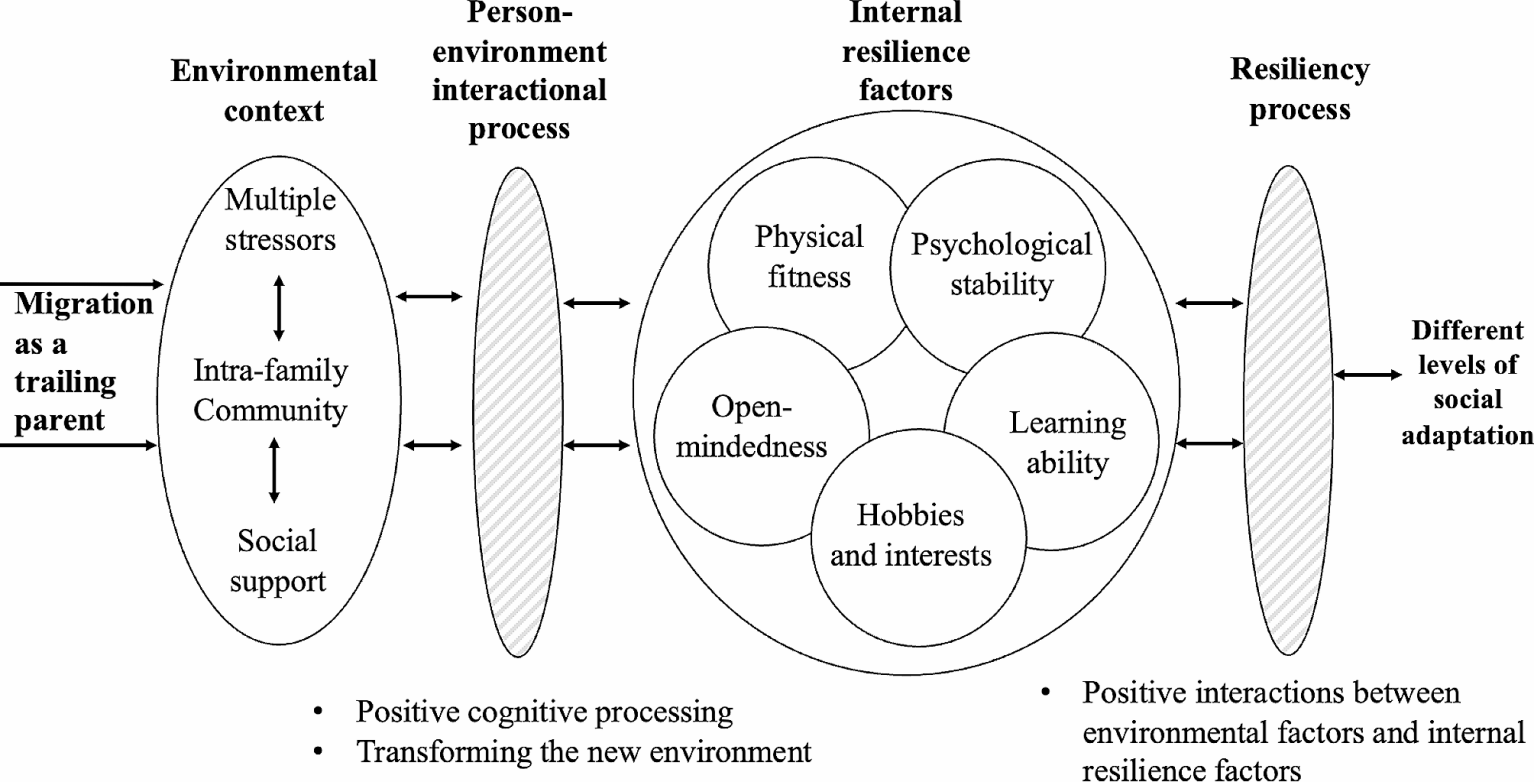
The dynamic social adaptation process of the trailing parents

Figure 1 summarizes the dynamics of social adaptation for the study participants, based on Kumpfer’s resilience framework and the findings in previous parts. Social adaptation as a trailing parent was a gradually unfolding process involving various factors and elements, including the challenge, the environmental context, the person-environment interaction process, internal resilience factors and the resilience process.

As trailing parents, the study participants faced multiple stressors at both the intra-family and community levels. They interacted with the new challenging environment, attempted to process positive cognitions about the stressors, and positively transformed the new environment. Within this person-environment interaction process, their internal resilience characteristics enabled them to cope better with stressors and change, and further promoted positive cognition and action to transform the community and intra-family environment. The resilience factors identified in this study of trailing parents included physical fitness, psychological stability, open-mindedness, learning ability and personal hobbies and interests. These factors could be strengthened by increasing support from family, friends and neighbors. The resiliency process is an integral part of the person-environment interaction process. If positively enacted, it would emphasize the interactions between environmental factors and individual internal resilience factors, which in turn would further contribute to the creation of a virtuous ecosystem, helping the trailing parents to achieve different levels of social adaptation.

## Discussion

Trailing parents are a special group that has emerged in the context of China’s rapid social change and urbanization. They may be more vulnerable than their urban and rural counterparts as they face the double dilemma of mobility and ageing [8, 28]. They live with a constant conflict between the old and the new environment and are unable to fully integrate into either. In this case, they are more dependent on their adult children and also struggle to find their identity in changing contexts.

Despite their large numbers, this unique group has received little attention. In most studies, their experiences are typically interpreted within the discussion of either the ‘older population’ or the ‘migrant population’ [29, 30]. Meanwhile, previous studies have paid most attention to highlighting the difficulties faced by this group of people [8, 13], but few studies have focused on how they cope with change and achieve some degree of successful social adaptation [12]. Using Kumpfer’s resilience framework, this study is one of the first attempts to provide an integrated view of the social adaptation process of trailing parents.

Kumpfer’s resilience framework begins with the identification of stressors that may activate one’s resilience characteristics. This study, similar to others [6, 15, 31], found that trailing parents faced both family and community stressors. At the family level, there was a strong tension between the identities of ‘caregiver’ and ‘parent’. Their adult children expect them to take on multiple responsibilities as dedicated caregivers, while they want to be listened to and respected as parents [32]. This has exacerbated the conflict between the generations and their nostalgia [9, 33]. At the community level, trailing parents are challenged by new lifestyles, values and social codes, but are restricted or isolated from both formal and informal social support systems [7, 28], making them feel like ‘outsiders’.

Resilience theory highlights the good qualities that motivate people to engage in positive cognitive processing and take actions to transform the environment. Trailing parents with physical fitness, psychological stability, open-mindedness, learning ability and some hobbies are more likely to be self-motivated to take steps to adapt. These characteristics may be inherent but cannot be interpreted as a static state [34]. Instead, these characteristics are dynamically shaped by the interaction between the individual and environmental systems [35]. In other words, people with these characteristics are more likely to start a virtuous circle, but those without them can also be nurtured.

Consistent with other studies [12, 13, 36], this study suggests that social support is of paramount importance in fostering resilience characteristics and facilitating social adaptation for trailing parents. As newcomers, people may experience loneliness, displacement and even emotional problems. Timely family support can alleviate these negative emotions and facilitate the development of social networks. Once they have successfully made new friends, trailing parents are less dependent on family members. At this stage, their homogenized social network can provide information, life skills and companionship to help them integrate better.

It should be noted that family support can also hinder the social adaptation process of the trailing parents if it is too strong. The older generation may become accustomed to relying on their help and refuse to build social networks and obtain resources in other ways. This can exhaust the younger generation and increase family conflict. So, as good supporters, family members should also learn another lesson about ‘letting go’.

Compared with older migrants who have obtained a local hukou, the psychological well-being of trailing parents who have only changed residence is poorer [37]. As a result of hukou policy in China, trailing parents face institutional barriers in accessing local public services and lack formal social support. In particular, we found that the accessibility and affordability of healthcare had a significant impact on their sense of belonging to the cities. Their migration status may affect the reimbursement process, thereby increasing their feelings of insecurity and deprivation [30, 38]. The trailing parents are in a state of confinement within the urban space. The lack of formal support conversely highlighted the importance of informal support from family and friends.

The plight of trailing parents has yet to become a clear public policy issue in China. At this stage, sound and practical interventions to help activate or nurture resilience characteristics may be of paramount importance in accelerating the social adaptation process and thereby improving their quality of life [39, 40]. This study contributes to extending Kumpfer’s resilience framework to trailing parents and highlights several principles for intervention. At the household level, small family routines that show emotional support are important to encourage the trailing parents to engage in positive cognitive processing and to try new things. Meanwhile, given that cognitive differences between generations cannot be avoided, it is wise for the adult children to help connect their parents with other older adults, making peer support to become the core emotional support for them. For urban communities, the institutional barriers to accessing social welfare cannot be quickly eradicated due to hukou policies, targeted support activities such as family-focused education, health lectures and volunteer activities can also help trailing parents build social networks and strengthen mutual support.

This study has some typical limitations common to qualitative studies. Firstly, the study findings were mainly based on interviews, in which there may be a Hawthorne effect [41]. Participants may have subjectively created a more positive and optimistic image because they realized they were being researched. Secondly, this study is based on people who were willing to participate and we approached them in public. Active participation in interviews is in itself a sign of good social adaptation. Trailing parents, whom we cannot approach, may have very different experiences. Thirdly, the living conditions of trailing parents are complex, involving family relationships, economic status, social support and many other aspects. Despite facing housing problems, the participants in this study had relatively good economic conditions because their adult children were doing well in the cities. Those with children of lower economic status may also face economic hardship, which was not addressed in this study.

A single qualitative study is not sufficient to fully capture the complexity of the social adaptation phenomenon of trailing parents in China. While this study provides a positive perspective for understanding the social adaptation process of trailing parents, their plight in urban life should not be neglected or downplayed. They have not only made an important contribution to their families, but also to the prosperity of the city by relieving the burden of young people. Their unique contribution to society should be recognized in the context of China’s massive urbanization. Future public policy should take responsibility for promoting a more inclusive social environment for them. Studies should also continue to examine the needs of trailing parents and take proactive measures to improve their social adaptation and quality of life.

## Conclusions

The social adaptation and psychological well-being of the trailing parents is a big challenge for China in promoting policies of new urbanization and active ageing. This qualitative study, based on Kumpfer’s resilience framework, found that although the study participants faced multiple intra-family and community stressors, they were not passive in the new context. Their resilience characteristics could be activated and even nurtured in the face of these challenges. Those with resilience characteristics such as physical fitness, psychological stability and learning ability were able to make positive cognitive and behavioral adjustments, which in turn further strengthened the resilience process. To further promote the social adaptation of trailing parents, interventions should focus on increasing their social support network.

## Data Availability

The datasets used and analyzed in this study are available from the corresponding author on reasonable request.

## References

[1] Cheng M, Duan C. The changing trends of internal migration and urbanization in China: new evidence from the seventh National Population Census. China Popul. Dev. Stud. 2021;5:275–295.

[2] Liu T, Wang J (2020). Bringing city size in understanding the permanent settlement intention of rural–urban migrants in China. Popul Space Place.

[3] Huang X, Zhao B, Liu Y, Xue D (2020). Belonging to a place: an analysis of the perceptions of rural-to-urban migrants in China. Geogr Rev.

[4] Li H, Chen K, Yan L, Yu L, Zhu Y (2023). Citizenization of rural migrants in China’s new urbanization: the roles of hukou system reform and rural land marketization. Cities.

[5] Zhao L, Liang C, Gu D (2021). Mobile social media use and trailing parents’ life satisfaction: social capital and social integration perspective. Int J Aging Hum Dev.

[6] Ruan Y, Wang D, Li D. Influence of Neighborhood-based identity and social participation on the Social Integration of the drifting Elderly. Journal of Health and Social Care in the Community. 2023;1:2101202.

[7] Fu Y, Lin W, Yang Y, Du R, Gao D (2021). Analysis of diverse factors influencing the health status as well as medical and health service utilization in the floating elderly of China. BMC Health Serv Res.

[8] Wang JJ, Lai DW (2022). Mental health of older migrants migrating along with adult children in China: a systematic review. Ageing Soc.

[9] Qi X (2018). Floating grandparents: rethinking family obligation and intergenerational support. Int Sociol.

[10] Zhu H (2021). Analysis on the Operation of Community Integration Project for Elderly migrants–the case of Fangshan District in Beijing. Int J Social Sci Educ Res.

[11] Gu X (2022). Sacrifice and indebtedness: the intergenerational contract in Chinese rural migrant families. J Fam Issues.

[12] Ruan Y, Zhu D, Lu J (2019). Social adaptation and adaptation pressure among the drifting elderly in China: a qualitative study in Shanghai. Int J Health Plann Manag.

[13] He X, Zhang F, Zhao H, Li J. How migration in later life shapes their quality of life: a qualitative investigation of the well-being of the drifting elderly in China. Soc Indic Res. 2020:1–25.

[14] Wu M, Peng C, Chen Y, Yuan M, Zhao M, Wang C (2020). Nurses’ perceptions of factors influencing elder Self-neglect: a qualitative study. Asian Nurs Res.

[15] Tang D, Xie L (2023). Whose migration matters? The role of migration in social networks and mental health among rural older adults in China. Ageing Soc.

[16] Xie J, Liao J, Zhang J, Gu J (2020). Association between rural-to-urban migration and the cognitive aging trajectories of older Chinese adults: results from a prospective cohort analysis. BMC Geriatr.

[17] Lyu J, Wang X, Fan DX. Ageing in the context of accompanying migration: a leisure stress coping perspective. Leisure Stud. 2024;43(2):311–326.

[18] Kumpfer KL. Factors and processes contributing to resilience: the resilience framework. Resilience and development: positive life adaptations. 2002:179–224.

[19] Guo H, Zhou R, Li M, Zhang S, Yi H, Wang L (2022). The use of Kumpfer’s resilience framework in understanding the breastfeeding experience of employed mothers after returning to work: a qualitative study in China. Int Breastfeed J.

[20] Zhang Z, Stein KF, Norton SA, Flannery MA (2023). An analysis and evaluation of kumpfer’s resilience framework. Adv Nurs Sci.

[21] Ma M, Gao R, Wang Q, Qi M, Pi Y, Wang T. Family adaptability and cohesion and the subjective well-being of parents of children with disabilities: the mediating role of coping style and resilience. Curr Psychol. 2023;42(22):19065–19075.

[22] Johnson JL, Adkins D, Chauvin S (2020). A review of the quality indicators of rigor in qualitative research. Am J Pharm Educ.

[23] Hennink M, Kaiser BN (2022). Sample sizes for saturation in qualitative research: a systematic review of empirical tests. Soc Sci Med.

[24] Braun V, Clarke V (2021). To saturate or not to saturate? Questioning data saturation as a useful concept for thematic analysis and sample-size rationales. Qualitative Res Sport Exerc Health.

[25] Phillippi J, Lauderdale J (2018). A guide to field notes for qualitative research: context and conversation. Qual Health Res.

[26] Jonsen K, Jehn KA (2009). Using triangulation to validate themes in qualitative studies. Qualitative Res Organ Management: Int J.

[27] Williams EN, Morrow SL (2009). Achieving trustworthiness in qualitative research: a pan-paradigmatic perspective. Psychother Res.

[28] Li J, Rose N (2017). Urban social exclusion and mental health of China’s rural-urban migrants–A review and call for research. Health Place.

[29] Chen F, Zheng M, Xu J, Hall BJ, Pan Y, Ling L (2022). Impact of migration status on incidence of depression in the middle-aged and elderly population in China: exploring healthy migrant and salmon bias hypotheses from a mental health perspective. J Affect Disord.

[30] Dou X, Liu Y (2017). Elderly migration in China: types, patterns, and determinants. J Appl Gerontol.

[31] Guo, Liu J, Xu L, Mao W, Chi I (2018). Intergenerational relationships and psychological well-being of Chinese older adults with migrant children: does internal or international migration make a difference?. J Fam Issues.

[32] Ruan Y, Zhu D (2021). Association of Chinese drifting elderly’s intergenerational support satisfaction with expectation: a mixed method study in Shanghai. Int J Health Plann Manag.

[33] Yan S, Deng R, Hou Y, Zhang L, Zhang W, Yao J. A latent class analysis of intergenerational relationships among the Elderly migrants in Nanjing, China. Psychol Res Behav Manage. 2023:1221–1232. 10.2147/PRBM.S404869 PMC10120831 37089819

[34] Vella S-LC, Pai NB (2019). A theoretical review of psychological resilience: defining resilience and resilience research over the decades. Archives Med Health Sci.

[35] Barasa E, Mbau R, Gilson L (2018). What is resilience and how can it be nurtured? A systematic review of empirical literature on organizational resilience. Int J Health Policy Manage.

[36] Wang J, Pang M, Kong F (2023). Association between self-reported oral health and life satisfaction among China’s migrant elderly following children: the mediating effect of social support. Front Public Health.

[37] Zhang NJ, Vanhoutte B (2021). The relationship between rural to urban migration in China and risk of depression in later life: an investigation of life course effects. Soc Sci Med.

[38] Han J, Meng Y (2019). Institutional differences and geographical disparity: the impact of medical insurance on the equity of health services utilization by the floating elderly population-evidence from China. Int J Equity Health.

[39] Zheng W, Huang Y, Fu Y (2020). Mediating effects of psychological resilience on life satisfaction among older adults: a cross-sectional study in China. Health Soc Care Commun.

[40] Kong L-N, Zhang N, Yuan C, Yu Z-Y, Yuan W, Zhang G-L (2021). Relationship of social support and health-related quality of life among migrant older adults: the mediating role of psychological resilience. Geriatr Nurs.

[41] Oswald D, Sherratt F, Smith S (2014). Handling the Hawthorne effect: the challenges surrounding a participant observer. Rev Social Stud.

